# The Role of Oral Cavity Biofilm on Metallic Biomaterial Surface Destruction–Corrosion and Friction Aspects

**DOI:** 10.3390/ijms19030743

**Published:** 2018-03-06

**Authors:** Joanna Mystkowska, Katarzyna Niemirowicz-Laskowska, Dawid Łysik, Grażyna Tokajuk, Jan R. Dąbrowski, Robert Bucki

**Affiliations:** 1 Department of Materials Engineering and Production, Faculty of Mechanical Engineering, Bialystok University of Technology, Wiejska 45C, 15-351 Bialystok, Poland; lysik.dawid@gmail.com (D.Ł.); j.dabrowski@pb.edu.pl (J.R.D.); 2 Department of Microbiological and Nanobiomedical Engineering, Medical University of Bialystok, Mickiewicza 2C, 15-222 Bialystok, Poland; katia146@wp.pl (K.N.-L.); buckirobert@gmail.com (R.B.); 3 Department of Integrated Dentistry, Medical University of Bialystok, M. Sklodowskiej-Curie 24a, 15-276 Bialystok, Poland; grazyna.t1@gmail.com

**Keywords:** biofilm, oral cavity, biocorrosion, metallic biomaterial, friction

## Abstract

Metallic biomaterials in the oral cavity are exposed to many factors such as saliva, bacterial microflora, food, temperature fluctuations, and mechanical forces. Extreme conditions present in the oral cavity affect biomaterial exploitation and significantly reduce its biofunctionality, limiting the time of exploitation stability. We mainly refer to friction, corrosion, and biocorrosion processes. Saliva plays an important role and is responsible for lubrication and biofilm formation as a transporter of nutrients for microorganisms. The presence of metallic elements in the oral cavity may lead to the formation of electro-galvanic cells and, as a result, may induce corrosion. Transitional microorganisms such as sulfate-reducing bacteria may also be present among the metabolic microflora in the oral cavity, which can induce biological corrosion. Microorganisms that form a biofilm locally change the conditions on the surface of biomaterials and contribute to the intensification of the biocorrosion processes. These processes may enhance allergy to metals, inflammation, or cancer development. On the other hand, the presence of saliva and biofilm may significantly reduce friction and wear on enamel as well as on biomaterials. This work summarizes data on the influence of saliva and oral biofilms on the destruction of metallic biomaterials.

## 1. Introduction

**Figure 1:**
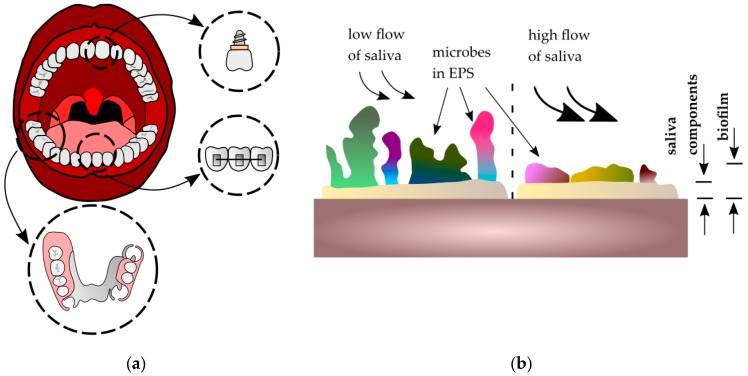
Biofilm on metallic biomaterials in the oral cavity: (**a**) applications of metallic biomaterials in the oral cavity; (**b**) influence of salivary flow on biofilm formation, where EPS—extracellular polymeric substances.

The application of biomaterials in the oral cavity has increased due to esthetic, surgical, and biofunctional reasons, mainly as elements of dental prosthetics, screws, implants [1,2,3,4], and orthodontic materials [5,6,7]. These appliances are placed in the oral environment and are subjected to many factors such as saliva, food, temperature fluctuations, masticatory forces, or appliance loading [8]. The dynamic development of new biomaterials has promoted advances in dental prosthetics and orthodontics. Modern dental materials include a wide group of polymers, ceramics, composites, metals, and their alloys. Despite enormous progress in the field of material engineering and the gradual displacement of metallic materials by polymers and ceramics, it is still difficult to find materials with better mechanical properties than metals (even in terms of tensile strength or fracture toughness). Among the metallic biomaterials used are noble metal alloys, austenitic steels, titanium, and cobalt alloys [9]. The environment of the oral cavity impacts metallic biomaterials and significantly limits the life and reliable functioning of dental materials. Saliva plays an important role and creates a liquid environment in the oral cavity. Its chemical composition (organic and inorganic substances) gives it lubrication, buffering, and antimicrobial properties [10,11]. Among the components responsible for maintaining homeostasis are mucins, which have the ability to adhere to the oral mucosa (so-called mucoadhesion). As a consequence, saliva covers the surface of the teeth, tongue, mucous membranes of the oral cavity, and the surfaces of biomaterials with a film thickness of 70–100 μm [12,13]. Saliva plays a protective function and is therefore responsible for lubrication processes. It also plays an important role in biofilm formation on the surface of biomaterials (Figure 1a). Biofilm is defined as a surface film composed of adsorbed organic and inorganic saliva components that are colonized with microorganisms (bacteria, fungi) in extracellular polymeric substances (EPS), covering all surfaces in the oral cavity [14].

The biofilm formation process leads to a change of oral cavity parameters, such as electrolytic concentration, pH, or oxygen levels [15]. The composition and structure of surface biofilm depend on the biomaterial’s localization. Moreover, the flow of saliva is also an important factor which may change the quantity and quality of biofilm. During saliva flow, the components of EPS can be changed, which may influence the adhesiveness of the biofilm to the surface. Biomaterials exposed to a high flow of saliva (where high shear forces occur) are less susceptible to biofilm formation [16] (Figure 1b). On the other hand, surfaces with locally high nutrient content for microorganisms are more susceptible to colonization—for example, between denture elements. The presence of biofilm on dental metallic biomaterials in the oral cavity is related to numerous processes of its surface destruction, such as corrosion and friction [17,18,19,20]. In the case of the second phenomenon (friction), the wear process of teeth and biomaterials is observed. The majority of reports [21,22,23] that examined the mechanisms of wear destruction in the oral cavity concentrate on titanium and its alloys because of their popularity due to perfect biocompatibility and good mechanical properties [24]. They are also characterized by less susceptibility to corrosion, as their surface is covered by a passive oxide layer (mostly TiO_2_ and Al_2_O_3_).

In this review, we briefly summarize the influence of biofilm on the surface destruction of metallic biomaterials (especially corrosion and friction) and highlight recent advances in the methods of controlling biofilm growth.

## 2. Microbial Flora and Biofilm Formation in Oral Environments

The oral environment is the richest and most varied in terms of the presence of microorganisms. Within the oral cavity, there are several distinct environments in which different microbial communities are present [25,26,27]. Recently published review reports indicate that more than 700 bacterial species have been identified by culture-independent approaches in the human oral cavity while over 250 of them have been described and named [28]. However, it is established (Table 1) that the oral microbiota is not only composed of numerous kinds of bacteria, but also of other kinds of microorganisms including ultra-small bacteria belonging to the “candidate phyla radiation (CPR) group” as well as fungi, phages, and viruses [28,29].

**Figure 2:**
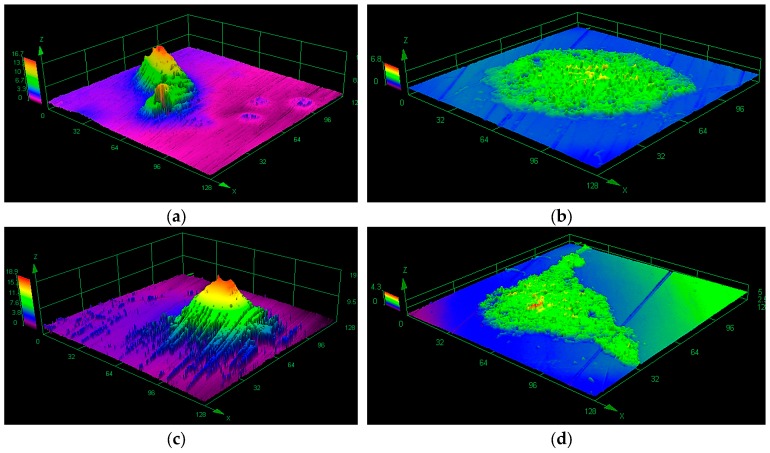
Biofilm on 316LV steel visualized by a confocal laser scanning microscope: (**a**) *Streptococcus mutans*, (**b**) *Candida albicans*, (**c**,**d**) *Streptococcus mutans* + *Candida albicans* (one-species and mixed-species biofilm was formed over one month in stationary conditions on the surface of steel 316LV).

There is a constant and transient microflora whose quantitative and qualitative composition depends on many factors such as oral hygiene, nutrition (including composition and consistency of food), population, tooth extraction, prosthetic restorations, dental and periodontal diseases, metabolic and cancer diseases, and dental treatments [30,31]. The main groups of bacteria in a healthy oral cavity are *Streptococcus* spp. that colonize the oxygen-rich areas of the oral cavity. Among them, the most common bacteria are *Streptococcus sanguinis*, *S. mutans*, *S. mitis*, *S. salivarius*, *S. pneumoniae*, *and S. milleri* [32]. *S. sanguinis* has a capacity to bind directly to oral surfaces and functions as a chain for the attachment of a variety of other oral microorganisms which colonize the tooth, form dental plaque, and contribute to caries and periodontal disease. Among the anaerobic bacteria that most often occur in inaccessible areas of the mouth can be the so-called sulfate-reducing bacteria (SRB). Their oral distribution is related to the oxidative potential that exists in the environment. Pathogenic yeasts of *Candida* spp., especially *Candida albicans*, are quite common in the oral cavity. They are found on the surfaces of austenitic steel 316LV (Figure 2), on teeth and elements of dental prosthetics, orthodontic devices, dental plaque, and cavities [33,34,35,36].

In such a biologically diverse oral environment and in the presence of numerous organic and inorganic saliva components, a biofilm is created on the surface of teeth and dental materials. The process of its creation proceeds at the interface, where saliva plays an important role. Present in saliva, glycoproteins and phosphoproteins, such as mucins and proline-rich proteins (histatins, statherins), adhere to the bacteria-free surfaces of teeth, oral mucosa, and biomaterials through ionic, van der Waals, and hydrophobic interactions [37,38,39] in a highly selective process [40,41]. Adsorption of proteins electrochemically changes tooth and biomaterial surfaces, which mediates interactions with the microbe-rich oral environment. In effect, microorganisms interact directly with built-in film-forming molecules which have an influence on the further colonization of adsorbed microbes [42,43]. After adsorption of microorganisms to the surface of the pellicle, further, faster, adhesion of microorganisms together with glycoproteins and phosphoproteins is observed. With time, so-called dental plaque and denture plaque are formed [44].

An important role in the process of biofilm growth is performed by saliva, which is a transporter of nutrients for persistent microorganisms in root canals. On the other hand, saliva is also a carrier of antimicrobial compounds: lysozyme, lactoferrin, sialoperoxidase, histatin, statherin, and bacteriocin. It is, therefore, the main source of substances necessary for producing the extracellular matrix (EPS) which, together with bacteria, forms a biofilm. This matrix is like a scaffold for bacteria, enabling bacterial development and providing protection against the external environment [45,46]. Constant saliva flow makes colonization of the oral cavity difficult for microorganisms and, to some extent, ensures control of biofilm growth [47]. Mucins and other glycoproteins contribute to this process through aggregating bacteria into larger complexes and attaching them to the mucosal surface, blocking the adhesion of other bacteria. This process is part of a protective mechanism against pathogenic organisms [48].

The presence of biofilms on the surface of teeth, implants, and dentures can be characterized by negative or positive effects. Negatives include plaque formation, caries, bacterial and fungal infections, adverse effects on structural changes, and lowering of endurance parameters and intensification of biomaterial destruction processes. Yokoyama et al. [49] indicate an intensive hydrogen uptake by implantable titanium alloys in a biological environment. This leads to lower plasticity, changes in grain size and structure (grinding), and reduced fatigue strength.

The presence of biofilm in root canals is one of the reasons for failure of endodontic treatment [50,51]. Organisms living in biofilms gain many benefits since there is a synergistic impact of species that create these complex communities. The structure is much more resistant to antimicrobial agents and host defense mechanisms [52,53,54,55]. As a result, microorganisms integrate into the biofilm structure [56,57]. This mechanism also includes fungal cells, including *Candida albicans*, which are capable of forming a stable biofilm structure. The dosage of drugs used must be several times higher in order to inhibit further growth of the bacteria.

On the other hand, plaques protect tooth and biomaterial surfaces against mechanical injuries such as attrition, abrasion, chemical effects, and harmful effects of microorganisms living in the biofilm form [37,58,59,60].

## 3. Corrosion

There are not many data documenting the side effects of metals and alloys used in dentistry. Contemporary dentistry in interdisciplinary treatment (endodontics, periodontics, dental surgery, orthodontics, prosthetics) uses various noble, minor, and base alloys. As mentioned earlier, metallic elements present in the oral cavity in the form of, e.g., dental implants, screws, post-and-cores, inlays, onlays, metal crowns, metal–ceramic crowns, skeletal dentures, or acrylic dentures with metal braces undergo corrosion and biocorrosion and interact with each other. Corrosion of metallic biomaterials is a result of electro-galvanic cell formation and current flow in the oral cavity due to the presence of metals with different electrochemical potential [61]. The occurrence of this phenomenon depends on the type of alloys, technological processes used for their production, laboratory treatment (thermal, chemical, mechanical), and oral conditions [62,63]. Electrochemical treatment of metal elements causes greater corrosion resistance than only mechanical treatment. This is important in the clinically observed activity of the oral environment, which promotes the surface release or electrode polarization of metallic elements. The ions emitted in electrochemical processes pass into the saliva. The probability of occurrence of this phenomenon is increased by incorrect processing of elements in the laboratory and incorrect adjustment by the dentist. Damage or removal of the passive layer from the surface of the alloy can also occur during everyday activities such as chewing food or brushing teeth.

The saliva present in the oral cavity is an electrolyte that changes under various factors [64]. Change in pH is influenced by systemic factors such as the age of patients, stress, some diseases and general infections, oral medications, bacterial flora present in the oral cavity, bacterial and viral infections, amount of saliva (including daily cycle), and type of food intake. Saliva pH is different at sites near the outlet of the salivary glands.

In the oral cavity, along with saliva, there are also extracellular fluids (e.g., blood, gingival fluid) that form a closed circuit. Therefore, in the oral cavity, the metal is submerged in two different electrolytes containing different concentrations of the same metal. It is important to avoid close contact between various metal elements as well as between metal and mucous membranes because it affects the frequency of occurrence of galvanic currents in the oral cavity. Amalgam used for tooth restoration has a high tendency to react electrochemically and is able to interact with other metals in the oral cavity [65]. The occurrence of the potential difference also causes the flow of current between the gums, tongue, and mucous membranes [66]. If this phenomenon occurs over longer periods, it causes lesions known as electrometallosis, the symptoms of which are inflammatory changes of the tongue, mouth, periodontium, and mucous membranes. There is also an imbalance in salivary secretion and other local and general changes in the body. Subjective symptoms reported by patients include burning of the tongue and mucous membranes and a sense of a metallic taste. The described phenomena are the cause of changes in the oral mucosa, especially dangerous precancerous states, e.g., leukoplakia. This is a result of constant stimulation caused by galvanic currents leading to keratotic changes, inflammation, erosion, and ulcerations. The currents can also be caused by BMS (burning mouth syndrome) [67], which is a neuropathic pain. BMS is a chronic condition of various sensations and discomfort in the oral cavity without any visible changes on the mucous membrane. One of the possible causes of BMS may be micro-injuries of sensory nerve endings resulting in spontaneous discharges without stimulation of their endings.

The presence of biofilm on the surface of metallic materials can dramatically increase corrosion processes. Aerobic microorganisms intensify the formation of diverse oxygenation cells which promote the development of crack corrosion. This can lead to loosening and dysfunction of implants [68]. The corrosion of metals used in dental prosthetics adversely affects their biocompatibility and mechanical integrity with the tissues of the organism [69] and may enhance allergy to metals [70]. Corrosion processes cause toxic and allergic reactions in the human body, inflammation, and cancer development [19,71,72]. In addition, metal ions released during the corrosion of metallic dental structures [70] may enter the gastrointestinal tract and accumulate in the stomach, liver, kidneys, spleen, bones, lungs, brain, or mucous membranes [73,74]. This is a significant problem, but still poorly understood.

The influence of biofilm on corrosion processes is not well characterized. The authors of works [75,76] have analyzed the effect of mucin on corrosive characteristics of metallic biomaterials. It has been observed that proteins and glycoproteins in PBS solution act as corrosion inhibitors and thus limit the electrochemical degradation of titanium alloys [77].

The surfaces of dental prostheses are most often associated with bacteria such as *Streptococcus mutans*, *S. sobrinus*, *S. sanguis*, *S. oralis*, *S. milleri*, *S. salivarius*, *Staphylococcus aureus*, *S. epidermidis*, *Actinomyces israeli*, *A. neslundii*, *A. odontolyticus*, *A. viscosus*, *Lactobacillus* spp., *Propionibacterium* spp., and *Veillonella* spp. [78]. In prosthetic dentistry, removable denture users often suffer from chronic atrophic candidiasis. The growth and development of *Candida* spp. occurs on the surface of the prosthesis or plaque [79], most often at the point of contact of the denture with teeth and mucous membranes. The composition of bacterial biofilm on the denture surface is similar to that of the biofilm present on the surface of teeth. This problem concerns about 11–67% of denture users [33,80,81]. The increase of temperature, humidity, decreased oxygen availability, and insufficient buffer and salivary rinsing activity under the denture plate all contribute to the development of microorganisms.

## 4. Biocorrosion

Most corrosion tests of biomaterials are induced electrochemically. However, it should be noted that in the heterogeneous environment of the oral cavity [82,83,84], besides typical oral microflora, there may also be microorganisms responsible for microbiologically induced corrosion of metallic biomaterials (MIC, Microbiologically Influenced Corrosion) [85]. Taking the above into account, biocorrosion refers to the accelerated deterioration of metals owing to the presence of biofilms on their surfaces [66].

Available biocorrosion studies [68,86,87,88] mainly concern the effects of typical oral cavity bacteria *Streptococcus mutans* and *Streptococcus sanguis*. Kameda et al. [70] investigated the effect of these microorganisms on the corrosion resistance of metallic elements of orthodontic devices (stainless steel (SUS) and NiTi). Their studies have shown that microorganisms are able to induce corrosion. They observed biocorrosion on the surfaces of steel but not in the case of titanium alloy appliances. On the other hand, in Souza’s work [89] the results of electrochemical tests (polarization resistance of a passive titanium oxide film) carried out in the presence of *Streptococcus mutans* indicate that the bacteria have a negative effect on the corrosion resistance of titanium alloys.

Wilson [90] tried to explain the idea of corrosion induced by *Streptococcus sanguis*. In his opinion, the presence of bacteria on the metal surface leads to production of cathode/anode regions, resulting in corroding currents. This leads to the creation of a wide range of metabolic products, such as organic acids, which can react directly with the metal. Authors of other works [69,82,91] also confirmed that microbial corrosion occurred under a biofilm as cathode/anode reactions.

**Figure 3:**
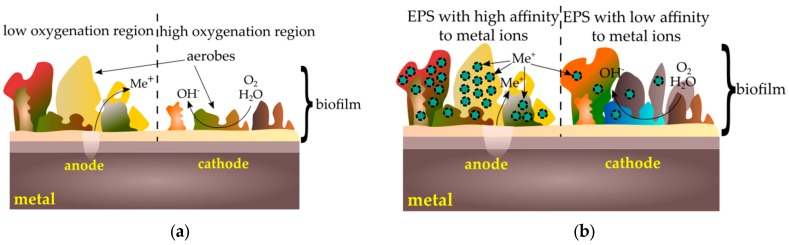
Corrosion cells caused by biofilm: (**a**) cells with varying degrees of oxygenation; (**b**) cells containing various concentrations of metal ions.

Corrosion cells occur when two areas are in contact with different concentrations of the same solution. There are three types of corrosive cells—cells with varying degrees of oxygenation, cells containing various concentrations of metal ions, and active–passive cells [92]. Cells with varying degrees of oxygenation occur when there is a difference in oxygen concentration between two areas. One of the factors causing the creation of such a mechanism is a heterogeneous layer of biofilm. Bacteria such as *Streptococcus mutans* use oxygen, so a nonuniform distribution of the microorganism layer causes differences in the oxygenation of particular areas: under the thicker layer of the biofilm the surface will behave anodically, under thinner—cathodically. The surface contact with a lower oxygen concentration will be the anode, while the surface in contact with a higher oxygen concentration—the cathode (Figure 3a). Another factor causing the formation of cells with different oxygenation is the simultaneous occurrence of aerobic and anaerobic organisms. Under aerobic bacteria colonies, the metal surface will be an anode, under the anaerobic colonies—a cathode. Diversification of oxygen concentration also results in the formation of heterogeneous precipitation structures dense on the edges and loose in the middle, which intensifies differences in oxygenation in particular areas. Due to the dense layer of corrosion products, oxygen diffusion is low, while where products are loose, oxygen passes more easily and diffusion is higher [92].

Corrosion microcells may occur due to differences in the concentration of metal ions on the surface. Microbes colonizing the surface produce extracellular polysaccharides (EPS). The resulting polymer matrix contains various functional groups capable of binding metal ions to a greater or lesser extent [92,93]. Areas under low-affinity colonies will, therefore, behave like anodes and areas under colonies with high affinity for metal behave like cathodes (Figure 3b).

**Figure 4:**
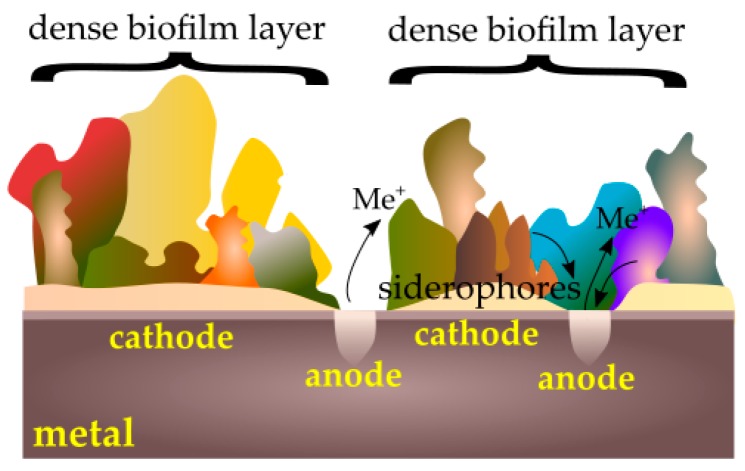
Passive–active cells caused by a biofilm.

Microorganisms can also accelerate the corrosion of metals by creating passive–active cells (Figure 4). When a tightly packed biofilm layer is broken, active metal under the coating is exposed to a corrosive reaction. Microorganisms are also able to break the already formed passive layers via secreted substances such as siderophores that can dissolve these layers [92].

Many species of bacteria coexist symbiotically. Some of them produce metabolic by-products that can support the growth of other bacteria. A typical example is between aerobic sulfide-oxidizing bacteria and sulfate-reducing anaerobic bacteria. Bacteria oxidize sulfides to elemental sulfur, which settles on the surface. Under the layer of sulfur, there are ideal conditions for the development of anaerobic bacteria. 

A similar mechanism occurs in the areas of aerobic bacteria colonies, under which the oxygen-poor region is located.

Anaerobic bacteria are found in the mouth due to the presence of so-called transitional flora. These include anaerobic bacteria that reduce sulfates (SRB, Sulfate-Reducing Bacteria) [88,94,95], as *Desulfovibrio* (*vulgaris*, *desulfuricans*, *fairfieldensis*, *gigas*) or *Desulfotomaculum nigrificans* [94,96,97]. At these conditions as a result of complex biochemical reactions may be released H_2_, H_2_S, and FeS (as a strong local cathode) leading to metal degradation and, consequently, to the gradual destruction of biomaterials. It is generally accepted that SRB are known as major pathogens causing corrosive processes. However, two representatives of other anaerobic Gram-negative pathogens—*Fusobacterium nucleatum* and *Prevotella melaninogenica*—are responsible for producing butyric acid, carbon dioxide, and hydrogen during enzymatic degradation of saccharides [98].

**Figure 5:**
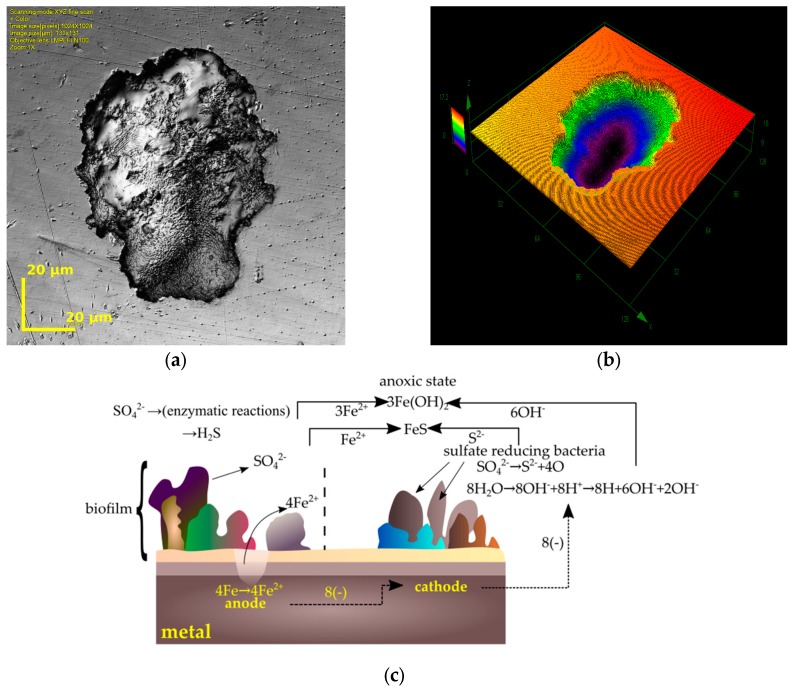
Corrosion induced by sulfate-reducing bacteria: (**a**,**b**) pits on 316LV found using a confocal scanning microscope (own research, 316LV tested in *D. nigrificans* environment during a 56 day period); (**c**) steel corrosion mechanism caused by sulfate-reducing bacteria [98].

The influence of sulfate-reducing bacteria (*D. nigrificans*) on the corrosion resistance of titanium (Ti–6Al–4V) and cobalt (Co–Cr–Mo) alloys and 316LV implantation steel was presented in studies [99,100]. It should be emphasized that more changes have been observed on the surface of 316LV steel (Figure 5a,b). As in other publications [84,101,102], traces of corrosion were not uniformly distributed on the surface of steel, and were most abundant in places where bacterial agglomerations were found.

Each of the corrosion pits had a surface area of a few μm^2^ (in the case of tested alloys) up to a few hundred μm^2^ (in the case of 316LV steel). With increased study time (from 28 to 56 days) the number of biocorrosion initialization products increased [99,100]. Similar data are presented in other papers [103,104]. The results of these microscopic observations show that titanium alloy is more resistant to sulfate-reducing bacteria, as is the case with typical electrochemical corrosion tests [105]. Gurappa [106], Hodgson [107], and Hsu [5] came to similar conclusions while emphasizing the high biocompatibility of titanium alloys.

The formation of corrosive changes caused by the presence of sulfate-reducing bacteria can be explained in many ways. Research presented in the paper of Lates et al. [94] demonstrates that SRB are responsible for the potential difference between the surface corroded by microorganisms and the area free from bacterial activity. This leads to the formation of local pitting on the metal surface, in the form of pitting corrosion wear [103,104]. In addition, their metabolic activity in combination with organic compounds can lead to the formation of aggressive corrosion products, e.g., organic acids [108].

In addition, there is also a reduction in grain size in the material to a depth of about 20 nm compared to the deeper layers of the biomaterial [99]. In Geesey’s work [109] the thickness of the oxide layer was in the range of 2–5 nm. Chen and Clayton [110] reported that the passive film on the steel surface may be affected by the negative influence of microbiologically induced sulfides and the removal of the alloying elements.

Biocorrosive products in the form of sulfides can become a cathode of a relatively large surface area, which further accelerates the processes of biocorrosion on metal surfaces. In the papers by Lata [103], Lopes [97], and Kumar [111], it was indicated that in the biocorrosion processes of steel in an anoxic environment, the metal surface acts as an anode in the reaction and is oxidized to produce Fe^2+^ ions. SRB reduce the sulfates to sulfide ions S^2−^, which react with Fe^2+^ ions to form a (dark) sludge of iron sulfide (II).

At the same time, in the cathode space, the same amount of hydrogen ions is produced that react with the hydroxyl groups from the aqueous medium [111]. It has also been found that the aqueous medium promotes the adhesion of sulfate-reducing bacteria to the metallic surface [96].

Hydrogen plays an important role in biocorrosive processes—as an electron donor in the sulfate reduction reaction. According to many studies [94,97,102,103,111] the total reduction of sulfates to sulfides is as follows (Figure 5c):

4Fe + SO_4_^2−^ + 4H_2_O → FeS + 3Fe(OH)_2_ + 2OH^−^

It is assumed that the presence of sulfides increases the susceptibility of the metallic biomaterial to corrosion, mainly due to increased material solubility, retardation repassivation, and weakening of the protective properties of the passive film surface. Under anoxic conditions, SRB play an important role in reducing the exploitation stability of metal implants. In addition, under these conditions, sulfate-reducing bacteria lead to the formation of hydrogen sulfide (H_2_S), a cell-toxic compound [94,112]:

SO_4_^2−^ + ATP (ATP sulfurylase) → APS + PPi (APS reductase) → AMP + SO_3_^2−^ (sulfite reductase) → H_2_S

Another mechanism describing the phenomenon of biocorrosion [113] indicates that *Desulfovibrio* spp. bacteria produce an enzyme called hydrogenase [69,114] which catalytically affects the reduction of sulfates of the corrosive environment and facilitates the use of hydrogen produced in the microcathodes of the corroded site. As a result, sulfate-derived oxygen is used for the oxidation of iron, leading to a gradual transition to iron oxide. This phenomenon is conducive to the aquatic environment of the oral cavity. The role of hydrogen in sulfate-reducing bacteria cells is described in the following reaction [115]:

NAD^+^ + H_2_ (hydrogenase activity) = NADH + H^+^

As a consequence of the above-mentioned mechanisms of microbiologically induced corrosion (MIC), the oxygen from sulfate reduction is consumed in iron oxidation, which over time passes into iron oxide Fe_2_O_3_. It has also been found that the action of corrosive bacteria causes a potential difference between the microbial and inert zones, leading to the formation of local corrosion pits.

A few scientific studies indicate the beneficial role of biofilm in the biocorrosion of metals [116,117]. One of the mechanisms that inhibit corrosion is the oxygen consumption of aerobic bacteria which stops the cathodic reaction. However, the area inhabited by this type of biofilm must be so homogeneous that no cell of variable oxygenation arises. Deprivation of oxygen in a particular area can reduce the secretion of polysaccharides in the biofilm matrix and reduce its cohesion forces. Alternative mechanisms inhibit the growth of harmful corrosive bacteria by the presence of compounds capable of antimicrobial activity. Other microbiological processes describe the removal of cathodic corrosion products due to bacterial activity (caused by diffusion of oxygen into the layer of biocorrosion products) or inhibition of growth of SRB due to the activity of antibacterial compounds produced by other bacteria present in the environment. Microorganisms can also produce biosurfactants that will reduce the adhesion of microbes to the surface. It should be also taken into consideration that the above-mentioned processes of forming protective layers on metallic surfaces (passive oxide layer or biofilm layer) are responsible for the formation of inhibitors with specific adhesive properties that prevent contact of corrosion products with the metal surface [100]. Counteraction against the corrosion process may prolong the exploitation time of biomaterials in the oral cavity.

## 5. Friction and Wear

The influence of biofilm on the processes of friction and wear of teeth and implant materials, including removable dental prostheses, is also an important topic. Friction is defined as the phenomenon which occurs between two moving relative surfaces and results in resistance of movement. Friction causes wear of materials which is manifested by loss of mass, volume, or thickness of the contacting surface. Different processes such as chewing, biting, swallowing, and speaking are performed in the oral cavity. Taking into account this heterogeneous phenomenon, there is difficulty in predicting and preventing tooth and biomaterial wear [118,119,120].

**Figure 6:**
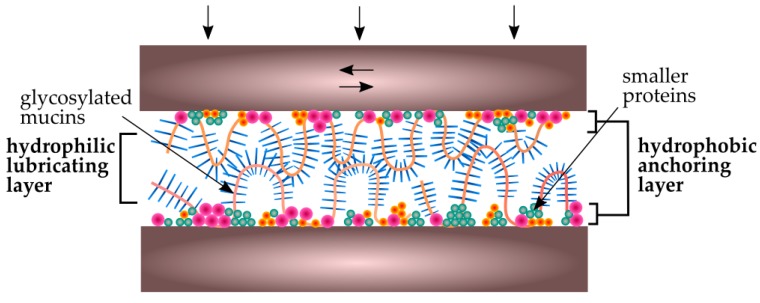
Schematic view of salivary conditioning film between biomaterial surfaces in the oral cavity [127].

The presence of biofilm on the surfaces of teeth can significantly reduce the friction and wear of the enamel as well as the biomaterials in contact with the teeth. The whole process occurs in the presence of saliva, which is a natural lubricant [121]. Its role is to reduce the friction between surfaces, which, in turn, reduces the material’s wear [122]. In vitro studies of friction coefficient (μ) [59,123,124] have shown that between different surfaces tested in saliva, μ values are 0.01–0.9, depending on the measurement conditions. The presence of mucin and other peptides (e.g., statherins) in saliva plays an important function in these processes, especially under boundary lubrication conditions. It firstly provides tooth protection under low occlusion load, e.g., during the chewing process, and also plays an important role during increased occlusion load, e.g., in bruxism [125]. The in vitro studies of the friction coefficient between hydrophobic PDMS (polydimethylsiloxane) surfaces tested in a mucin solution have shown that its values (μ ~ 0.1) are not as low as in the oral cavity (μ ~ 0.01) [126]. Studies of the friction coefficient between mica surfaces (hydrophobic and hydrophilic in different combinations) tested in purified statherins have shown that μ values are between 0.09–0.88. This means that the coefficient of friction depends essentially on the composition (i.e., interactions between low- and high-molecular-weight mucins, statherins, and proline-rich proteins), and in effect on the structure of the adsorbed film. In one study [127] it was confirmed that the structure of the layer adsorbed on hydrophobic surfaces is heterogeneous. The inner layer consists of low-molecular-weight proteins and non-glycosylated MUC5B mucin fragments [128]. The outer layer is less regular and is formed of hydrophilic, highly glycosylated chains of these mucins. Yakubov et al. [129] have confirmed that the key to such good lubrication is the macroscopic coverage of saliva-contaminated surfaces and the synergistic interaction of mucins and low-molecular-weight proteins. As is shown in Figure 6, the inner layer (hydrophobic anchoring layer) is more compact and is responsible for wear resistance, while the lubricating properties provide an external, strongly hydrated mucin (hydrophilic lubricating layer) [127].

An important factor in the lubrication process is the concentration of mucins in saliva. Due to the fact that the adsorption process depends on mucin concentration, saliva dilution at high extents serves to produce a thinner biofilm layer and, thus, its lubricating properties are reduced [130].

However, in some specific conditions, another phenomenon occurs. In this case, when acidic substances are present in the oral cavity or mechanical injuries occur, the enamel surface is more hydrophilic. As a result, the hydrophobic parts of the mucins responsible for antiwear layer formation are not adsorbed on its surface [38]. In effect, microorganisms start to adsorb simultaneously or even faster than saliva’s glycoproteins. In this case, the biofilm layer of mucins is not formed and there is no tooth or biomaterial protection against friction and wear.

Friction properties of a biomaterial’s surface biofilm are related to its viscoelastic properties. High viscosity and elasticity cause that biofilm to behave like a lubricant, which decreases the friction between the surfaces in contact. Depending on the shear force, the biofilm may exhibit reversible elastic or irreversible viscous behavior [18]. With increased shear forces, the biofilm structure changes phase from an elastic-solid (it reaches a breaking point), after which it begins to behave like a viscous fluid [131,132].

Apart from viscoelasticity, the biofilm is characterized also by specific mechanical properties, mainly formed by the EPS matrix which constitutes more than 90% of the dry biofilm mass [133]. The structure of the extracellular matrix depends on the physicochemical interactions, mainly electrostatic and van der Waals forces and hydrogen bonds. Any defects in those cohesion forces may result in biofilm separation and removal from the biomaterial structure (so-called breaking strength), resulting in an increase in the friction force. This process is highly dependent on boundary interactions between the biofilm and biomaterial surface.

Taking the above into account, many studies have been undertaken. Souza [17] examined the morphology of titanium covered by different biofilms before, during, and after the friction process. The countersample to titanium was aluminium oxide and the kinematic pair was tested in artificial saliva. A reduction of the friction coefficient was observed in the case of a titanium surface covered with biofilm. In another paper from Souza [18], the corrosion of titanium covered by a biofilm during the friction process was examined. The friction coefficient was reduced and, simultaneously, a decrease of corrosion resistance was observed. In a previous work [134], the influence of LPS (lipopolysaccharides) on tribocorrosion wear of titanium and its alloys was evaluated. The results indicate a higher ion exchange between titanium and saliva and lower corrosion resistance of the biomaterials. The presence of LPS during friction increased the weight loss and roughness of the surface. Also, Messer et al. [135] observed a reduction of titanium alloy corrosion in the presence of blood, monocytic cells, and a solution with glucose. Besides the reduction of corrosion resistance, an increase in surface roughness for titanium alloys was also observed. A similar result was obtained in the presence of hydrogen peroxide, which is released in the oral cavity by bacteria and leukocytes during inflammation (i.e., caused by mechanical injuries) [135]. In another work [136], the influence of stimulated inflammatory conditions on corrosion resistance and surface roughness of commercially pure titanium and stainless steel was examined. The results showed a decrease in the corrosion resistance of titanium (greater than stainless steel) and an increase of surface roughness and free surface energy of samples after treatment.

**Figure 7:**
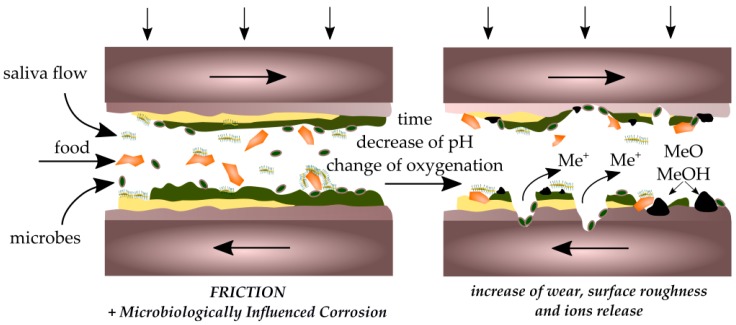
Scheme of the interaction of corrosion and tribological processes in the destruction of biomaterials.

The discussed studies indicate a reduction in the coefficient of friction and wear due to the presence of biofilms. At the same time, in most of them, a decrease in the corrosion resistance of the material was observed. The presence of biofilms in the oral cavity has a greater potential for the formation of corrosive microcells than for the inhibition of corrosion processes. This can lead to a situation in which the biofilm layer ruptures due to high shear forces and contact with surface roughness protrusions. The corroded material surface will be exposed, which will accelerate further material wear due to friction (Figure 7).

## 6. The Role of Saliva in Biofilm Growth

As mentioned earlier, saliva plays an important role in controlling the growth of biofilm. The reduction of its secretion due to disease processes and drug use causes an uncontrolled growth of pathogenic bacteria and fungi in the oral cavity. This leads not only to inflammation of soft tissues and increased susceptibility to caries but also to the acceleration of processes of wear and corrosion of biomaterials. Artificial saliva with an appropriate composition may play a major role in preventing these processes. Directions for improving the performance of such preparations should take into account their chemical modification. This is mainly about biologically active compounds with antimicrobial properties that will be able to prevent bacterial adhesion to the surface of medical materials and the consequent formation of bacterial biofilm. Proper selection of artificial saliva components has the potential to influence the kinetics and mechanism of biofilm formation on metallic biomaterials.

## 7. Conclusions

Corrosion and wear processes depend on biomaterial surface biofilm formation. The composition and flow of saliva plays an important role. In the biofilm structure there are biological and physicochemical reactions which influence the properties of the biomaterial structure, i.e., surface roughness. Biofilm plays also an important role as a natural lubrication layer, which reduces wear of biomaterials.

## Figures and Tables

**Table 1:** Oral microbiome [28,29].

**Oral Bacteria Microbiome**	
Saliva	*Actinobacteria*, *Bacteroides*, *Firmicutes*, *Fusobacteria*, *Proteobacteria*, *Spirochaetes*, *TM7* (The Human Microbiome Consortium)
Dental plaque	*Firmicutes*, *Actinobacteria*
Oral mucosa	*Streptococcus salivarius*, *Rothia mucilaginosa*, *Eubacterium strain FTB41*
**Oral Bacteria Related to Oral Diseases**	
Dental caries	*Streptococcus*, *Veillonella*, *Actinomyces*, *Granulicatella*, *Leptotrichia*, *Thiomonas*, *Bifidobacterium*, *Prevotella*, *Lactobacillus*, *Propionibacterium*, *Pseudoramibacter*, *Selenomonas*
Periapical infections (Periapical periodontitis, root canal infection)	*Proteobacteria*, *Firmicutes*, *Bacteroidetes*, *Fusobacteria*, *Actinobacteria*, *Olsenella uli*, *Prevotella baroniae*, *Porphyromonas endodontalis*, *Fusobacterium nucleatum*, *Tannerella forsythia*, *Propionibacterium propionicum*, *Porphyromonas gingivalis*, *Prevotella intermedia*, *Prevotella oralis*, *Parvimonas micra*, *Porphyromonas endodontalis*, *Fusobacterium nucleatum*, *Tannerella forsythia*
Periodontal diseases (Gingivitis, Periodontitis)	*Actinomycetes*, *Capnocytophaga*, *Campylobacter*, *Eikenella*, *Fusobacterium*, *Prevotella*, *Porphyromonas gingivalis*, *Treponema denticola*, *Tannerella forsythia*, *Bacteroidetes* spp., *Eubacterium saphenum*, *Porphyromonas endodontalis*, *Prevotella denticola*, *Parvimonas micra*, *Peptostreptococcus* spp., *Filifactor alocis*, *Desulfobulbus* spp., *Dialister* spp., *Synergistetes*
Halitosis	*Solobacterium moorei*, *Atopobium parvulum*, *Eubacterium sulci*
